# Characteristics of Nonmotor Symptoms in Progressive Supranuclear Palsy

**DOI:** 10.1155/2016/9730319

**Published:** 2016-06-05

**Authors:** Ruwei Ou, Wei Song, Qianqian Wei, Ke Chen, Bei Cao, Yanbing Hou, Bi Zhao, Huifang Shang

**Affiliations:** Department of Neurology, West China Hospital, Sichuan University, Chengdu, Sichuan 610041, China

## Abstract

*Objectives*. To explore the clinical correlates of nonmotor symptoms (NMS) in progressive supranuclear palsy (PSP) and their differences from healthy controls and patients with Parkinson's disease (PD).* Methods*. Twenty-seven PSP patients, 27 age- and gender-matched healthy controls (HC), and 27 age- and gender-matched PD patients were included for this case-control study. NMS were assessed using the Nonmotor Symptoms Scale (NMSS, including 9 domains).* Results*. All PSP patients reported NMS. The frequency and severity of “sleep/fatigue,” “mood/apathy,” “attention/memory,” “gastrointestinal,” “sexual dysfunction,” and “miscellaneous” domains in PSP group were significantly higher than those in HC group (*P* < 0.05). The frequency of “mood/apathy,” “attention/memory,” and “sexual dysfunction” domains and the severity of “attention/memory” and “gastrointestinal” domains in PSP group were significantly higher than those in PD group (*P* < 0.05). The “attention/memory” domain in PSP had a significant but weak-to-moderate correlation with age (*R* = 0.387, *P* = 0.046) and onset age (*R* = 0.406, *P* = 0.036).* Conclusions*. NMS are common in PSP patients. Patients with PSP seem to be subjected to more frequent and severe specific NMS compared to healthy aging subjects and PD patients. Older PSP patients and late-onset patients are likely to be subjected to cognitive decline.

## 1. Introduction

Progressive supranuclear palsy (PSP) is one of the most common forms of neurodegenerative Parkinsonism after Parkinson's disease (PD), which can affect around 4.9/10000 inhabitants [1]. The incidence of PSP is close to 10% of the incidence of PD, making it more common than generally recognized [2]. PSP is characterized by some motor disturbances including early postural instability, backward falls, Parkinsonism, and vertical supranuclear gaze palsy [3], which may result in significant disability and have an impact on health-related quality of life (HRQoL) in patients with PSP [4]. In addition, some patients with PSP also display cognitive dysfunction, sleep disorders, and behavioral changes [5–7]. These symptoms are described as nonmotor symptoms (NMS), which has been widely reported in patients with PD [8]. NMS were also reported to affect the HRQoL of patients with PSP [9, 10], indicating that more attention should be paid to the management of these symptoms in PSP.

Several studies have examined a single NMS in patients with PSP. For example, the symptoms of rapid eye movement (REM) sleep behavior disorder (RBD) [11], depression [12], cognitive decline [6], and pain [13] have been found to exist in patients with PSP. However, very few studies have applied a specific rating scale to investigate the features of global NMS in PSP at present. The PRIAMO study [9] found that the most frequent NMS in PSP are gastrointestinal symptoms (80%) and fatigue (80%) by conducting the clusters (gastrointestinal symptoms, pain, urinary symptoms, cardiovascular autonomic symptoms, sleep disorders, fatigue, apathy, attention, skin disorders, psychiatric symptoms, respiratory symptoms, and other symptoms) to evaluate the frequency of NMS. However, another study [10] found that the most frequent NMS in patients with PSP is attention/memory problem by applying the Nonmotor Symptoms Scale (NMSS) to assess the frequency of NMS.

No study at present can answer the question if the frequency and severity of NMS in PSP are different from healthy aging subjects. Therefore, the first aim of this study was to make a quantitative comparison of NMS between PSP patients and age- and gender-matched healthy controls (HC) to assess the burden of NMS in PSP. In addition, to further assist neurologists manage NMS in patients with Parkinsonism and improve therapeutic strategies, another quantitative comparison of NMS between PSP patients and age- and gender-matched PD patients was performed to identify the distinct patterns of NMS in PSP from PD. Finally, a correlation analysis was implemented to identify the clinical correlates of NMS in PSP.

## 2. Patients and Methods

A total of 27 patients with PSP from the Department of Neurology, West China Hospital of Sichuan University, between 2013 and 2015 were continuously enrolled for this case-control study. All participants came from neurological inpatient ward and the outpatient clinic. Another 27 age- and gender-matched (1 : 1 matched) HC and 27 age- and gender-matched (1 : 1 matched) patients with PD were enrolled as controls. Patients or HC who had the same gender and equal or approximate age were regarded as a pair. The PD patients were recruited from the same hospital, while the HC were collected from a community in the area of residence. The HC who had any neurological diseases by neurologists were ruled out from this study. PD or PSP patients who had any other neurological diseases apart from PD and PSP were also excluded from this study. This study was approved by the Ethics Committee of West China Hospital of Sichuan University. All participants signed informed consent. PSP were diagnosed according to the Clinical Diagnostic Criteria of National Institute of Neurological Disorders and Stroke and the Society for PSP (NINDS-SPSP) for clinically probable PSP [3]. In order to avoid excluding PSP-Parkinsonism (PSP-P) patients from the selection, the criterion “in the first year of the disease” was not applied. Patients with a diagnosis of PSP were classified as having Richardson's syndrome (RS) and PSP-P based on the accepted clinical criteria [14]. Patients fulfilling the NINDS-PSP criteria for probable PSP within the first two years of disease onset were classified as RS. Patients initially presenting with asymmetric symptoms and slight therapeutic response to levodopa, but who fulfilled the NINDS-PSP criteria within the first 5 years of disease onset, were classified as PSP-P. At the same time, PD patients were grouped into three subtypes including tremor-dominant, akinetic-rigid, and mixed based on the criteria described in a previous study [15]. Other types of atypical Parkinsonism including dementia with Lewy bodies (DLB), corticobasal degeneration (CBD), and multiple system atrophy (MSA) and secondary Parkinsonism, as well as patients with difficulty in completing this NMSS questionnaire (e.g. severe dysarthria), were excluded from the present study.

Clinical data including age, sex, age of onset, total levodopa equivalent daily dosage (LEDD), and disease duration were collected by trained neurologists through a standardized face-to-face interview. Age of onset in the study was considered as the time when first reported symptom was attributable to PSP or Parkinsonism. Disease duration was calculated from the time when motor symptoms began to the time when a patient was interviewed. The total LEDD (mg/day) was calculated from the LEDD for each anti-Parkinsonian drug, as previously suggested [16]. The diagnosis of PSP and the clinical data were checked by another neurologist specializing in movement disorders to ensure the accuracy of the data. The Unified PD Rating Scale (UPDRS) part III [17] and Hoehn and Yahr (H&Y) stage [18] were used to evaluate the severity of the diseases. The NMSS [19] including 9 domains (cardiovascular, sleep/fatigue, mood/apathy, perceptual problems/hallucinations, attention/memory, gastrointestinal, urinary, sexual dysfunction, and miscellaneous) was applied to assess the frequency and severity of NMS in the last one month. A patient who scored 1 or more in any item by the multiplying of frequency (1–4) and severity (0–3) indicates that the patient had this NMS. The frequency of each NMS was calculated from the percentage of patients who obtained scores ≥ 1 in each item and subdomain of NMSS. The severity of NMS was calculated by multiplying frequency (1–4) by severity (0–3). Higher score indicates more severe NMS. The assessments of UPDRS were conducted on a state of being “off” medication both in PD and in PSP.

### 2.1. Statistical Analyses

All analyses were performed using the Statistical Package for the Social Sciences (SPSS) version 19.0 for Windows. All statistical tests were two-tailed and *P* < 0.05 was considered statistically significant. Continuous data were presented as mean ± standard deviation (SD), while discontinuous data were presented as median values (quartile). Fisher's exact test was employed to analyze the differences in category data between different case-control groups. The Mann-Whitney *U* test was performed to compare the difference in ranked data (H&Y stage) between PSP group and PD group. The paired-sample *t*-test was conducted for the comparison of the differences in continuous data between different groups. Pearson's correlation or Spearman correlation coefficient was used to analyze the correlations between NMS and clinical variables in PSP. Correlation coefficients (*R* value) more than 0.65 were arbitrarily chosen as indicative of strong correlations, those less than 0.25 were considered indicative of negligible correlation, and those in between were taken as indicative of weak-to-moderate correlation.

## 3. Results

### 3.1. Demographic and Clinical Features of PSP, PD, and HC

This study included 27 PSP patients (male : female = 16 : 11) with mean age of 65.1 ± 8.4 years, mean disease duration of 3.6 ± 2.1 years, and mean onset age of 61.5 ± 8.5 years. A total of 20 PSP patients (74.1%) were classified as RS, and the remaining 7 patients (25.9%) were classified as PSP-P. Meanwhile, 2 PD patients (7.4%) were classified as tremor-dominant type, 16 PD patients (59.3%) were classified as akinetic-rigid type, and the remaining 9 patients (33.3%) were classified as mixed type. The demographic and clinical features of PSP patients, PD patients, and HC are listed in Table 1. Patients with PSP had significantly greater H&Y stage than patients with PD (*P* < 0.05). No significant differences in age, age of onset, disease duration, and mean UPDRS III score were found between patients with PSP and PD. Age was not different between PSP patients and HC.

### 3.2. NMS in PSP and HC

Comparison of the frequency of NMS and mean NMSS scores in the PSP patients and the HC subjects are presented in Table 2. All PSP patients (100%) referred at least one NMS. The “sleep/fatigue” (100%), “mood/apathy” (100%), and “attention/memory” (96.3%) were the three most commonly affected domains in the PSP patients. The frequencies of “sleep/fatigue,” “mood/apathy,” “attention/memory,” “gastrointestinal,” “sexual dysfunction,” and “miscellaneous” domains from NMSS in the PSP group were significantly higher than in the HC group (*P* < 0.05), whereas no differences were found in the total NMSS and the remaining domains of NMSS between the PSP and the HC groups. The total score of NMSS and the scores of “sleep/fatigue,” “mood/apathy,” “attention/memory,” “gastrointestinal,” “sexual dysfunction,” and “miscellaneous” domains from NMSS in the PSP group were significantly higher than in the HC group (*P* < 0.05), whereas no differences were found in the scores of the remaining domains from NMSS between the PSP and HC groups.

### 3.3. NMS in PSP and PD

Comparisons of the frequency of NMS and mean NMSS scores in the PSP patients and the PD patients are presented in Table 3. The frequency of “mood/apathy,” “attention/memory,” and “sexual dysfunction” domains from NMSS in the PSP group was significantly higher than in the PD group (*P* < 0.05), whereas no differences were found in the total NMSS and the remaining domains from NMSS between the PSP and the PD groups. The scores of “attention/memory” and “gastrointestinal” domains from NMSS in the PSP group were significantly higher than in the PD group (*P* < 0.05), whereas no differences were found in the total score of NMSS and the scores of the remaining domains from NMSS between the PSP and the PD groups.

### 3.4. Correlation between NMS and Clinical Variables in PSP

The correlations between NMSS scores and clinical variables in PSP patients are listed in Table 4. The score of “attention/memory” domain from NMSS in PSP patients had a significant but weak-to-moderate correlations with age (*R* = 0.387, *P* = 0.046) and age of onset (*R* = 0.406, *P* = 0.036), whereas no significant correlations were found between the scores of the remaining domains of NMSS and the clinical variables including disease duration and UPDRS III score.

## 4. Discussion

To the best of our knowledge, this is the first case-control study to compare the differences in the frequency and severity of NMS between PSP patients and healthy controls. In the current study, we found that all patients with PSP reported at least one NMS. In addition, the most common NMS were “sleep/fatigue,” “mood/apathy,” and “attention/memory” domains in the current study, but no significant differences were found in the frequency among these NMS (*P* > 0.05), indicating that the NMS of sleep disturbances, emotional symptoms, and cognitive decline are the most prevalent affected fields in PSP. This finding is consistent with a previous study on 12 PSP patients, which found that “attention/memory” symptom is the most affected filed in PSP [10]. Furthermore, we found that PSP patients were subjected to more frequent and severe NMS particularly “sleep/fatigue,” “mood/apathy,” “attention/memory,” “gastrointestinal,” “sexual dysfunction,” and “miscellaneous” symptoms compared to healthy controls in the current study, suggesting that these NMS are characteristically existent in PSP. However, the frequency and severity of the remaining domains of NMSS were not different between PSP patients and healthy aging subjects, indicating that “cardiovascular,” “perceptual problems/hallucinations,” and “urinary” symptoms are not unique in PSP.

Sleep disturbance was observed in our PSP patients. This finding has been verified by two previous studies [7, 20] that used polysomnography (PSG) or quantitative electroencephalogram (EEG) to evaluate sleep architecture in PSP, respectively. PSP patients were reported to have significantly decreased total sleep time, sleep spindles, and REM sleep, along with a significant increase in the number and duration of nocturnal awakenings. A previous study [21] found that sleep in PSP is more severely impaired than in PD by polysomnography, but no difference in sleep was found by using PD Sleep Scale (PDSS) questionnaire, indicating that PSP patients rate their sleep problems differently from PD patients. The preferential degeneration of the pontine tegmental nuclei in the brainstem, particularly the pedunculopontine tegmental nuclei (PPTg), may explain these findings [22]. PPTg is thought to be important for the generation of REM sleep, and the connections between the PPTg and the reticular nuclei of the thalamus are important for generating spindles. Furthermore, as one kind of sleep disorders, RBD has been reported in 38–75% of neurological disorders particularly in neurodegenerative disorders characterized by intraneuronal deposition of *α*-synuclein (synucleinopathies, such as PD, MSA, and DLB), but it is rare in tauopathies such as PSP [23]. Our present study did not focus on this issue. In order to clarify the vital role of sleep disorder in PSP, further special studies on RBD should be conducted.

Neuropsychiatric symptoms were observed in our PSP patients. This finding is consistent with a case-control study [24] and several cross-sectional studies [5, 12], which found that neuropsychiatric symptoms including apathy, depression, and anxiety are common in PSP. Moreover, two previous case reports [25, 26] found that depression can be the first sign of PSP. These neuropsychiatric changes can be explained by frontal lobe dysfunction in PSP, which has been verified in several studies [27, 28]. In addition, some previous imaging studies found that white matter tract damage of corpus callosum, right superior longitudinal and uncinate fasciculi [29], and atrophy of putamen [30] are involved in behavioral deficits in PSP.

Cognitive deficits were observed in our PSP patients, which are consistent with the reports of several studies [6, 31]. Cholinergic deficits are thought to underlie the cognitive impairment in PSP [32]. In addition, an imaging study [33] found that cerebellar atrophy might play a role in the pathogenesis of cognitive dysfunction in patients with PSP due to a disruption of its modulation on executive functions. Another imaging study [29] indicated that white matter tract damage of corpus callosum, right superior longitudinal and inferior longitudinal fasciculus, and left uncinate may contribute to the executive deficits in PSP.

High frequency of gastrointestinal as well as sexual dysfunctions was observed in our PSP patients compared to healthy controls, which are consistent with a previous case-control study on 32 PSP patients and 27 HC [34]. Evidences on the findings are limited. Cerebral cortical and subcortical cholinergic deficits may play a role in the pathogenesis of autonomic dysfunction in PSP [35].

Miscellaneous problems such as pain, olfactory disorder, and weight reduction were found to be more frequent and more severe in our patients with PSP compared to healthy aging subjects. Pain in PSP has been reported in a case report [13]. Degeneration of the descending inhibitory control system within the brainstem in PSP might lead to increased experimental pain sensitivity while frontal cortical deterioration may alter self-estimation of pain [36]. Our current finding that olfactory disorder was more severe in PSP patients than in healthy controls is also consistent with a previous study [37], which applied the University of Pennsylvania Smell Identification Test to identify hyposmia. Neurofibrillary tangles and tau accumulation in the rhinencephalon may help to explain this phenomenon [37].

Significantly, the “mood/apathy,” “attention/memory,” and “sexual dysfunction” symptoms were found more frequently in our PSP patients than in PD patients, indicating that neurologists should pay more attention to these symptoms upob managing PSP patients. Furthermore, the “attention/memory” and “gastrointestinal” symptoms were found to be more severe in our PSP patients than in PD patients, indicating cognitive dysfunction and gastrointestinal problems may be a significant NMS to differentiate PSP from PD.

Finally, attention/memory problems in PSP were found to be weakly to moderately associated with age and age of onset, suggesting that older PSP patients and late-onset patients are likely to be subjected to difficulty with cognition. These findings are consistent with the accepted views that cognitive dysfunction is more universal in the general elderly population and increases with age [38]. Therefore, the underlying mechanism of the correlations identified in the present study is not easy to be explained. Further perspective study focusing on the cognitive assessment in PSP will help confirming this issue. In addition, no correlations were found between NMS and UPDRS III scores or disease duration in PSP, suggesting that NMS in PSP may be not interfered by disease severity or duration. We suspect that NMS may be an isolated symptom that exists in PSP. However, this conclusion should be considered cautiously, since the small sample size in the current study was not sufficient to make a definite conclusion.

Several limitations should be acknowledged. (1) The validation of NMSS in PSP needs to be further verified. (2) Small sample size in the current study cannot represent all of the disease course and all types of the disease. (3) The present results will be interfered with by the nonneurological conditions since the healthy controls were enrolled from the community. (4) The frequency and severity of NMS can be affected by some undisclosed drugs application. (5) The assessment of NMS can be influenced by some uncontrolled interference factors since patients with severe psychological and cognitive dysfunctions were not excluded from the present study. (6) There is a lack of objective confirmation of the diagnosis of PSP. (7) The present results cannot reflect the impact of NMS on quality of life of patients. Further longitudinal study with larger sample size will help confirming our findings.

## 5. Conclusions

NMS were common in PSP. Patients with PSP are subjected to more frequent and severe NMS compared to healthy controls. Older patients and late-onset PSP patients are likely to be subjected to cognitive dysfunction. Some NMS, particularly in the mood/apathy, attention/memory, and sexual dysfunction symptoms and the attention/memory and gastrointestinal symptoms, are more common in PSP than in PD patients. Recognizing these symptoms will be beneficial in helping neurologists manage patients with PSP.

## Figures and Tables

**Table 1:** Demographic and clinical features of PSP, PD, and HC.

Characteristics	PSP	PD	*P* value	PSP	HC	*P* value
Male : female	16 : 11	16 : 11	—	16 : 11	16 : 11	—
Age	65.1 ± 8.4	65.3 ± 8.4	0.367	65.1 ± 8.4	65.6 ± 8.4	0.301
Onset age	61.5 ± 8.5	61.7 ± 8.6	0.727	—	—	—
Disease duration	3.6 ± 2.1	3.6 ± 1.9	0.747	—	—	—
UPDRS III	32.7 ± 7.9	32.1 ± 11.3	0.572	—	—	—
H&Y stage	3 (1)	2.5 (1)	<0.001^*∗*^	—	—	—
LEDD	325.76 ± 262.99	351.85 ± 256.54	0.714	—	—	—

PSP: progressive supranuclear palsy. PD: Parkinson's disease. HC: healthy controls. UPDRS: Unified PD Rating Scale. ^*∗*^Significant difference.

**Table 2:** Frequency of NMS and mean NMSS scores in PSP and HC.

NMSS domain	Frequency of NMS		*P* value	NMSS score		*P* value
	PSP, *n* (%)	HC, *n* (%)		PSP, mean ± SD	HC, mean ± SD	
Cardiovascular	5 (18.5)	5 (18.5)	1.000	1.2 ± 3.5	0.3 ± 0.6	0.497
Sleep/fatigue	27 (100)	16 (59.3)	<0.001^*∗*^	12.4 ± 9.5	3.0 ± 4.5	<0.001^*∗*^
Mood/apathy	27 (100)	3 (11.1)	<0.001^*∗*^	16.6 ± 15.4	0.9 ± 3.1	<0.001^*∗*^
Perceptual problems/hallucinations	4 (14.8)	2 (7.4)	0.669	0.2 ± 0.6	0.2 ± 0.8	0.705
Attention/memory	26 (96.3)	16 (59.3)	0.002^*∗*^	7.1 ± 7.4	1.7 ± 2.5	<0.001^*∗*^
Gastrointestinal	23 (85.2)	8 (29.6)	<0.001^*∗*^	7.1 ± 7.0	1.1 ± 2.1	<0.001^*∗*^
Urinary	20 (74.1)	18 (66.7)	0.766	4.7 ± 5.5	3.0 ± 3.3	0.288
Sexual dysfunction	25 (92.6)	13 (48.1)	0.001^*∗*^	6.5 ± 6.7	1.8 ± 1.9	0.001^*∗*^
Miscellaneous	23 (85.2)	10 (37.0)	0.001^*∗*^	5.7 ± 5.6	1.8 ± 3.9	0.002^*∗*^
Total	27 (100)	25 (92.6)	0.491	61.6 ± 31.5	13.7 ± 11.8	<0.001^*∗*^

NMS: nonmotor symptoms. NMSS: Nonmotor Symptoms Scale. PSP: progressive supranuclear palsy. HC: healthy control. SD: standard deviation. ^*∗*^Significant difference.

**Table 3:** Frequency of NMS and mean NMSS scores in PSP and PD.

NMSS domain	Frequency of NMS		*P* value	NMSS score		*P* value
	PSP, *n* (%)	PD, *n* (%)		PSP, mean ± SD	PD, mean ± SD	
Cardiovascular	5 (18.5)	5 (18.5)	1.000	1.2 ± 3.5	1.2 ± 3.0	0.916
Sleep/fatigue	27 (100)	24 (88.9)	0.236	12.4 ± 9.5	11.1 ± 9.5	0.509
Mood/apathy	27 (100)	18 (66.7)	0.002^*∗*^	16.6 ± 15.4	11.1 ± 15.7	0.114
Perceptual problems/hallucinations	4 (14.8)	5 (18.5)	1.000	0.2 ± 0.6	0.3 ± 0.9	0.546
Attention/memory	26 (96.3)	16 (59.3)	0.002^*∗*^	7.1 ± 7.4	3.0 ± 3.8	0.018^*∗*^
Gastrointestinal	23 (40.4)	17 (63.0)	0.119	7.1 ± 7.0	3.2 ± 4.0	0.041^*∗*^
Urinary	20 (74.1)	17 (63.0)	0.559	4.7 ± 5.5	5.9 ± 7.2	0.537
Sexual dysfunction	25 (92.6)	15 (55.6)	0.004^*∗*^	6.5 ± 6.7	4.7 ± 7.5	0.148
Miscellaneous	23 (85.2)	21 (77.8)	0.728	5.7 ± 5.6	6.5 ± 5.3	0.753
Total	27 (100)	26 (96.3)	1.000	61.6 ± 31.5	47.0 ± 31.0	0.064

NMS: nonmotor symptoms. NMSS: Nonmotor Symptoms Scale. PSP: progressive supranuclear palsy. PD: Parkinson's disease. SD: standard deviation. ^*∗*^Significant difference.

**Table 4:** Correlations between NMSS scores and clinical variables in PSP.

	Age		Onset age		Disease duration		UPDRS III	
	*R*	*P* value	*R*	*P* value	*R*	*P* value	*R*	*P* value
Cardiovascular	−0.246	0.216	−0.238	0.232	−0.011	0.956	0.295	0.136
Sleep/fatigue	0.109	0.589	0.130	0.518	−0.098	0.627	0.048	0.811
Mood/apathy	0.154	0.444	0.129	0.521	0.087	0.666	−0.065	0.746
Perceptual problems/hallucinations	0.033	0.871	−0.039	0.848	0.292	0.140	−0.188	0.349
Attention/memory	0.387	0.046^*∗*^	0.406	0.036^*∗*^	0.104	0.607	0.083	0.683
Gastrointestinal	0.058	0.775	0.037	0.854	0.076	0.708	0.099	0.623
Urinary	−0.137	0.496	−0.096	0.635	−0.160	0.427	−0.104	0.607
Sexual dysfunction	−0.110	0.586	−0.075	0.708	−0.130	0.518	0.027	0.894
Miscellaneous	0.087	0.667	0.094	0.642	−0.033	0.869	0.129	0.520
Total	−0.028	0.889	−0.027	0.892	−0.003	0.988	0.063	0.753

NMSS: Nonmotor Symptoms Scale. PSP: progressive supranuclear palsy. UPDRS: Unified Parkinson's Disease Rating Scale. ^*∗*^Significant difference.
